# Effect of Layer Thickness and Heat Treatment on Microstructure and Mechanical Properties of Alloy 625 Manufactured by Electron Beam Powder Bed Fusion

**DOI:** 10.3390/ma15217767

**Published:** 2022-11-03

**Authors:** Julio Cesar Diaz, Kurtis Watanabe, Aldo Rubio, Alex De La Cruz, Dana Godinez, Shadman T. Nabil, Lawrence E. Murr, Ryan B. Wicker, Edel Arrieta, Francisco Medina

**Affiliations:** 1 W.M. Keck Center for 3D Innovation, The University of Texas at El Paso, El Paso, TX 79968, USA; 2 Department of Aerospace and Mechanical Engineering, The University of Texas at El Paso, El Paso, TX 79968, USA

**Keywords:** electron beam powder bed fusion (EBPBF), Inconel 625, microstructure and mechanical properties, layer thickness effects, heat treatment, duplex grain structure, grain boundary carbides

## Abstract

This research program investigated the effects of layer thickness (50 µm and 100 µm) on the microstructure and mechanical properties of electron beam powder bed fusion (EBPBF) additive manufacturing of Inconel 625 alloy. The as-built 50 µm and 100 µm layer thickness components were also heat treated at temperatures above 1100 °C which produced a recrystallized grain structure containing annealing twins in the 50 µm layer thickness components, and a duplex grain structure consisting of islands of very small equiaxed grains dispersed in a recrystallized, large-grain structure containing annealing twins. The heat-treated components of the microstructures and mechanical properties were compared with the as-built components in both the build direction (vertical) and perpendicular (horizontal) to the build direction. Vickers microindentation hardness (HV) values for the vertical and horizontal geometries averaged 227 and 220 for the as-built 50 µm and 100 µm layer components, respectively, and 185 and 282 for the corresponding heat-treated components. The yield stress values were 387 MPa and 365 MPa for the as-built horizontal and vertical 50 µm layer geometries, and 330 MPa and 340 MPa for the as-built 100 µm layer components. For the heat-treated 50 µm components, the yield stress values were 340 and 321 MPa for the horizontal and vertical geometries, and 581 and 489 MPa for the 100 µm layer components, respectively. The elongation for the 100 µm layer as-built horizontal components was 28% in contrast with 65% for the corresponding 100 µm heat-treated layer components, an increase of 132% for the duplex grain structure.

## 1. Introduction

Inconel 625 alloy, a nickel-based superalloy having high strength, ductility, corrosion resistance, and wear properties has found extensive applications in the aerospace, chemical, petrochemical, and various marine industries [1,2,3]. Over the past decade, significant advances have been made in the use of additive manufacturing techniques for producing Inconel 625 components, especially complex shapes involving heat exchanger components, turbine blades and parts, and chemical reactor parts. There have been a considerable number of investigations examining the microstructure and mechanical properties of both laser and electron beam powder bed fusion additive manufacturing of Inconel 625 alloy components in both the as-built and various post-process heat-treated conditions [4,5,6,7,8,9,10].

Inconel 625 is variously strengthened by heat treatment and aging above ~700 °C where gamma double prime (Ni_3_Nb) precipitation occurs, and in some cases by various carbides (MC, M_6_C, M_23_C_6_) for higher temperature heat treatment above 1000 °C, including HIP (hot isostatic pressing) [2,11]. While it has been shown that build parameters such as beam power, P, and power density, Q, as well as beam size, scan spacing, scan rate, and layer thickness can affect residual microstructure development such as columnar grains versus more equiaxed grain structures [12,13], there is a dearth of investigations addressing the effects of layer thickness (t) on residual microstructures and mechanical properties of additively manufactured metals and alloys. However, no studies of layer thickness effects have been conducted on electron beam powder bed fusion (EBPBF) of the Inconel 625 alloy. Layer thickness studies, especially those involving thicknesses above the general standard of 50 µm, can be a significant feature in large-volume component fabrication as a result of the increased build rate [13].

Only one EBPBF study has been conducted for Ti-6Al-4V layer thickness variations of 200–300 µm [14], and only one laser powder bed fusion study has been conducted for the Inconel 718 alloy, comparing components built with layer thicknesses of 30 µm and 50 µm. In this study, the size of the columnar dendritic cells in the build direction increased with layer thickness while the corresponding yield strength decreased [15]. LENS manufacturing of M300 maraging steel showed that increasing layer thickness increased porosity and degraded the residual mechanical properties [16].

The novelty of the present study is that it is the first to compare as-built microstructures and corresponding mechanical properties (including microindentation hardness and tensile properties) for electron beam powder bed fusion additively manufactured Inconel 625 alloy having different layer thicknesses of 50 µm and 100 µm. More importantly, these layer thickness components, examined in both the build direction and perpendicular to the build direction, were subsequently heat treated (including HIP) at temperatures above 1100 °C, and the associated microstructures and mechanical properties were compared with those for the as-built 50 µm and 100 µm layer components.

## 2. Materials and Methods

### 2.1. Powder Feedstock

**Figure 1:**
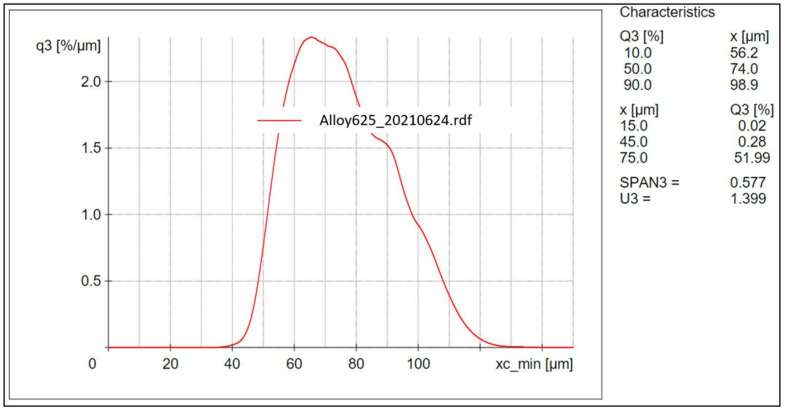
Particle size distribution graph.

Inconel 625 powder was purchased from Praxair Surface Technologies Inc (Indianapolis, IN, USA) (chemical composition shown in Table 1). The powder was manufactured through the gas atomization method. When characterizing the powder, the results for particle distribution, measured using a Retsch Camsizer X2 (Haan, Germany), showed that 10% of the powder measures less than 55.7 µm in diameter, 50% less than 75.9 µm in diameter, and 90% less than 101.1 µm in diameter as shown in Figure 1. Flowability and apparent density respectively resulted in 2.91 g/s and 4.16 g/cm^3^.

### 2.2. Electron Beam Powder Bed Fusion System

The machine used was an Arcam A2X (Mölndal, Sweden) with parameters as shown below in Table 2. Prior to the build, the outgassing temperature was 500 °C held for 30 min, followed by powder sintering at 950 °C held for 45 min. The same parameter values were used for both (50 µm vs. 100 µm) layer thicknesses, with the exception of repetitions (increased to 10) and average current (increased to 15 mA) during preheat 2 of the 100 µm layer thickness build which were intentionally changed to maintain build temperature; as more material was deposited, more energy density needed to be applied which was achieved by changing the aforementioned parameters (parameter development). These changes were closely monitored during the printing process by assuring no “smoking events” or “arc-trips” occurred during, which ensured a successful print and indicated the changed parameters functioned properly. Printing mostly with the same parameters for both layer thicknesses means that the previous layers re-melted by the 100 µm layer thickness are less than those re-melted by the 50 µm layer thickness. Consequently, the main build variable was the layer thickness (50 µm or 100 µm).

### 2.3. Heat Treatment

After manufacturing the samples, some of them were subjected to the following heat treatment:

1. Hot isostatic pressing (HIP) was carried out in an inert atmosphere at not less than 100 MPa within the range of 1120 to 1175 °C and held at that temperature within ± 15 °C for 240 ± 60 min and then cooled under an inert atmosphere as per ASTM 3301-18a.
2. Solution treatment at 1177 ± 3.9 °C for 60 min and argon gas fan cooled at a rate of air cooling or faster to 649 °C as per ASTM 7000.

This heat-treatment schedule was selected after examining the various precipitate temperatures, including carbides. The final HIP temperature was 1145 °C based on a minimum temperature of 1050 °C required for complete recrystallization in Inconel 625 for achieving a homogeneous, recrystallized grain structure. The solution temperature suppressed precipitate growth with the exception of carbides [2,3,13]. 

### 2.4. Microstructure Characterization

In the microstructure characterization phase, the printed specimens were sectioned along their X–Z, Y–Z, and X–Y planes and then mounted using epoxy to later be analyzed. The samples were then grinded and polished using an ATM SAPHIR 530 (Mammelzen, Germany) machine. Later, the samples were electro-etched in a solution containing 70 mL of phosphoric acid and 30 mL of water at room temperature using 5 volts for 30 s to 2 min. They were finally analyzed using an Olympus GX53 (Tokyo, Japan) microscope to determine the grain sizes which were calculated using the ASTM E-112 Standard Test Methods for Determining Average Grain Size. In some cases, direct, manual measurements were made.

### 2.5. Density Measurements

The density of the parts was taken using a gas displacement pycnometer system, Micrometrics AccuPyc II 1340 (Norcross, GA, USA), which works by pressurizing a small chamber with the specimen inside using helium gas and calculating the volume of the specimen while also taking its mass into account. This system then provides an average value of 10 density measurements.

### 2.6. Tensile Testing

**Figure 2:**
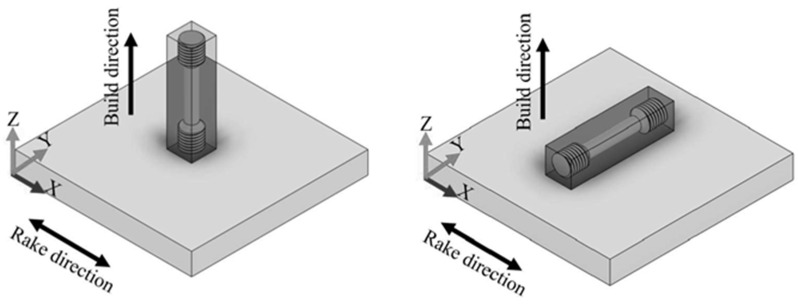
Specimen build directions.

Tensile coupons were machined from the additively manufactured parts following ASTM E8 Standard Test Methods for Tension Testing of Metallic Materials. Vertical and horizontal coupons were created, considering their build direction, as shown in Figure 2, in order to compare their mechanical performance by applying a uniaxial monotonic tensile load at a strain rate of 0.179 mm/min to each specimen. The specimen dimensions were 6 mm in diameter and 10 mm in length. Five tensile specimens per variant were tested; results show the average values of each variant and the standard deviation (SD).

### 2.7. Hardness Testing

Hardness testing was performed in a Struers Duramin-A300 (Copenhagen, Denmark) testing machine which makes micro indentations on the mounted samples (X–Z, Y–Z planes) and then reads the average value, reported in Vickers microindentation hardness (HV), using a 100 gf load. Five microindentations were made per sample to determine the average hardness value.

## 3. Results and Discussion

**Figure 3:**
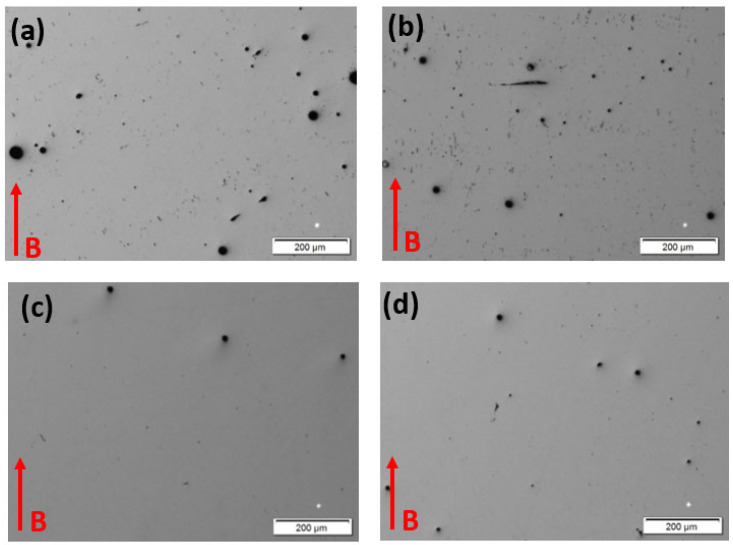
As-polished optical micrographs. (**a**) 50 μm AB; (**b**) 100 μm AB; (**c**) 50 μm HT; and (**d**) 100 μm HT. B denotes the build direction.

Figure 3 shows a comparative view of the polished and unetched as-built (AB) and heat-treated (HT) 50 µm and 100 µm layer Inconel 625 components, illustrating the residual porosity (black dots). It is observed that in the as-built 50 µm layer components, the pore sizes range from ~40 µm to 2 µm, while for the 100 µm layer components, the size range is ~25 µm to 2 µm; there was a higher small pore fraction at 100 µm layer thickness. The measured porosity for select areas was ~0.4% for both layer thicknesses. Correspondingly, for the heat-treated samples in Figure 3c,d, the range of pore sizes was ~15 µm to 2 µm, with a measured porosity of 0.1%. A total of 100 individual pore measurements were made in 3× magnified images. Since the measured pores were circular, the measured porosity represents the volume porosity. Densities for these layer thicknesses were 99.78% and 99.40% for Figure 3a,b, respectively, and 99.54% and 99.40% for Figure 3c,d, respectively. It is readily apparent in comparing Figure 3a,b with Figure 3c,d that the heat treatment (especially the HIP treatment) reduced the pore sizes and the overall porosity. The small porosities < 0.5% and densities > 99% are nominally better than those required for AM product development [17,18].

**Figure 4:**
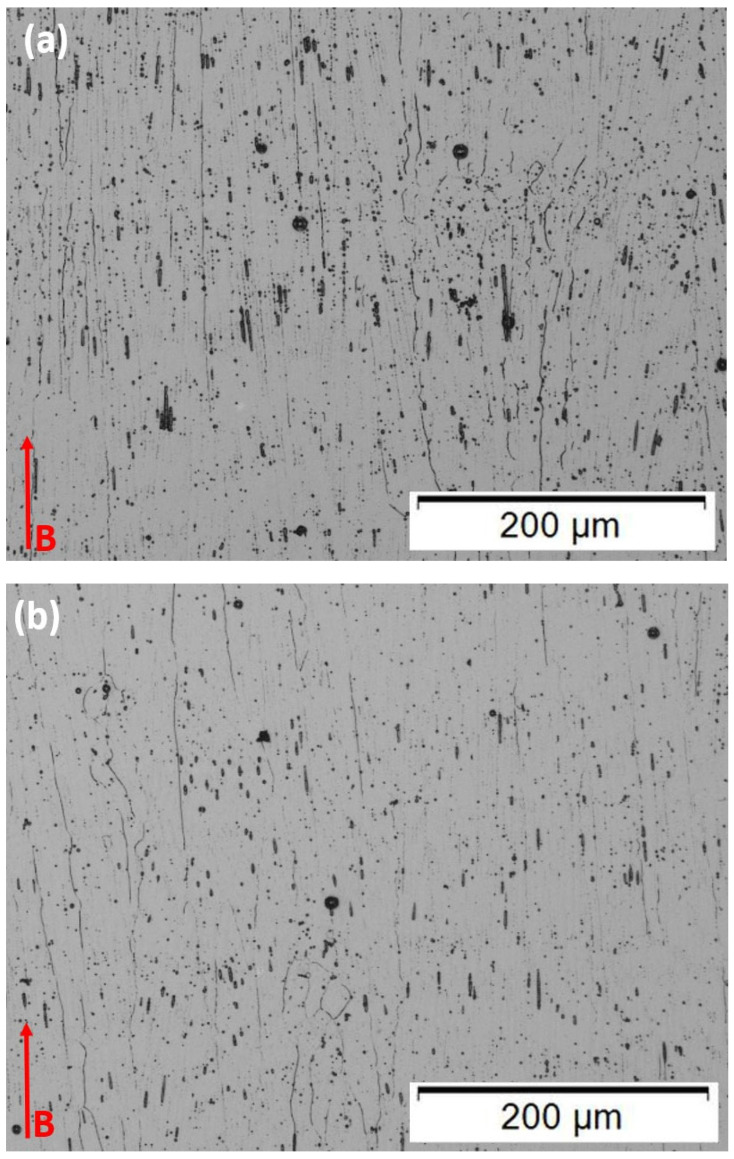
As-built samples viewed in the build plane. Build direction is shown at B. (**a**) 50 µm, layer thickness; (**b**) 100 µm layer thickness.

Figure 4 compares optical micrographs for the as-built microstructures for the 50 µm and 100 µm layer thicknesses in the horizontally oriented specimens (in the build plane; Figure 2) where B denotes the build direction. The microstructure consists of discontinuous precipitate columns spaced 2–3 microns, identified previously as gamma-double prime (Ni_3_Nb) [4,19]. This spacing is consistent with the beam scan melt pool dimension which has been shown to produce regular cubic arrays of this dimension in the X–Y plane perpendicular to the build direction [4,19]. However, it is apparent that the precipitate densities are noticeably greater in the 50 µm layer (Figure 4a). In addition, each layer thickness contains extended, columnar grains in the build direction, each having an average width of ~30 µm while the lengths vary from ~100–600 µm for the 50 µm layer components (Figure 4a), and 75–450 µm for the 100 µm layer components (Figure 4b). This difference is also reflected in the Vickers microindentation hardness (HV) measurements of HV 227 ± 12 for the 50 µm layer thickness components, and HV 220 ± 9 for the 100 µm layer thickness components. It is also notable that the layer thickness is very diffused (Figure 4), and virtually indistinguishable, especially since many columnar grains continue across many thicknesses as a consequence of epitaxial growth.

The difference in the microstructure in comparing Figure 4a,b is probably due in part to the smaller energy or power density, Q, which is half that for the 100 µm layer thickness components since Q is proportional to P/t [13], where P is the beam power and t is the layer thickness. Correspondingly, the layer cooling rate is also proportional to e^Q^ [13] and, therefore, declines by ~25% for the thicker layer fabricated components.

**Figure 5:**
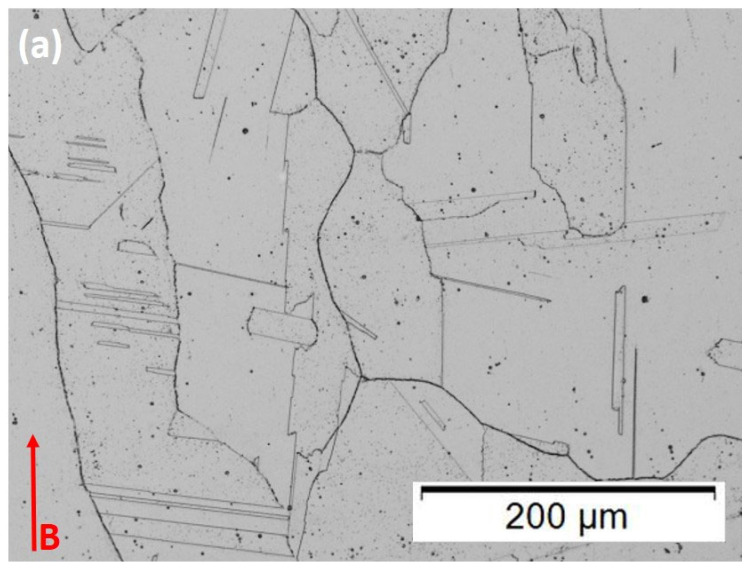

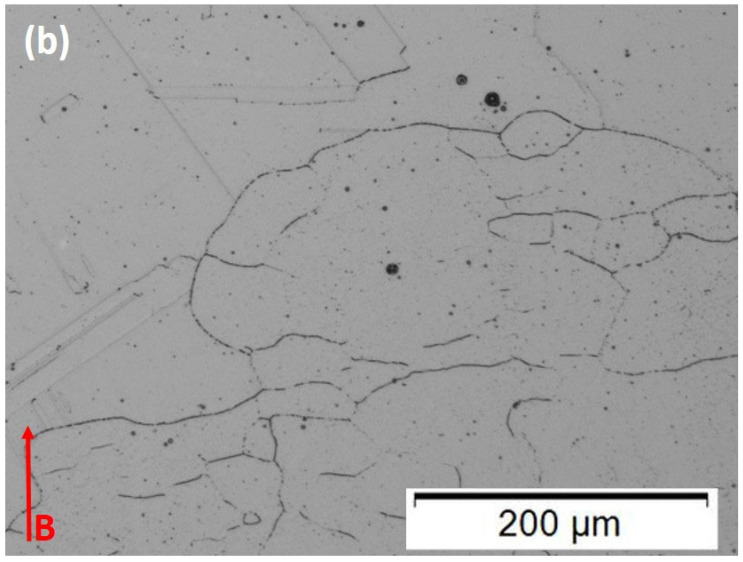
Heat-treated samples. (**a**) 50 µm layer thickness; (**b**) 100µm layer thickness. B denotes the build direction.

**Figure 6:**
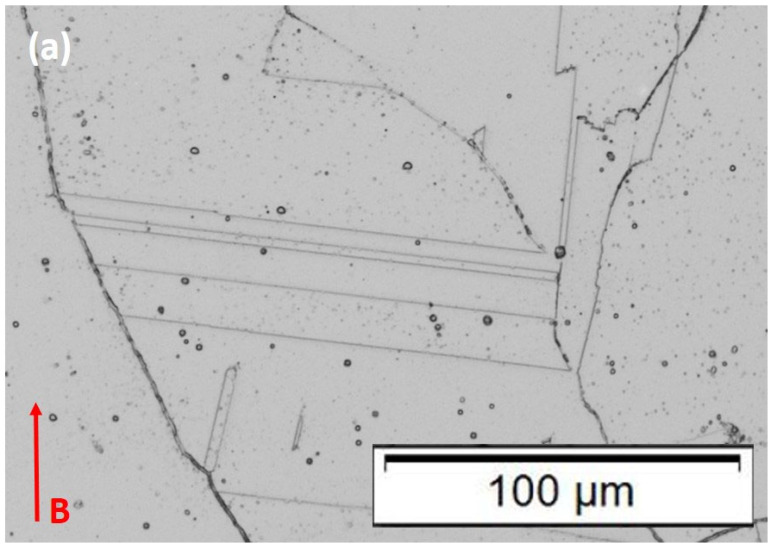

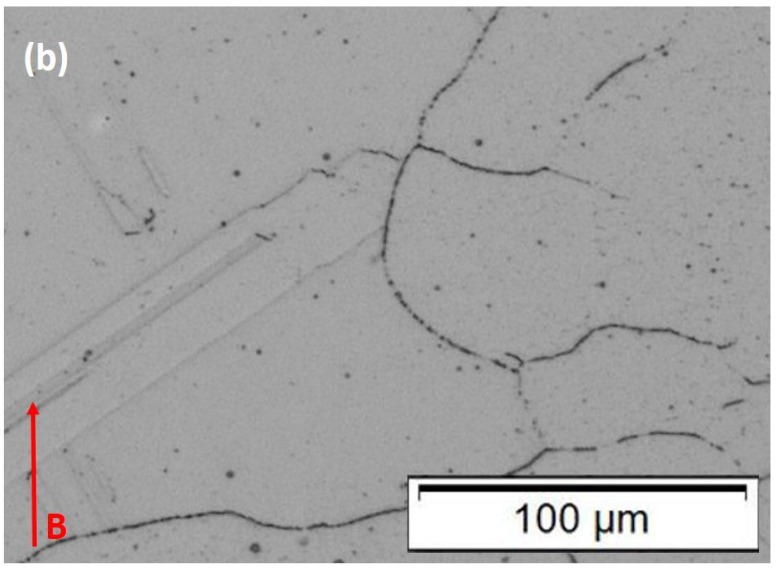
Heat-treated samples. Magnified images. (**a**) 50 µm, layer thickness; (**b**) 100 µm layer thickness. B denotes the build direction.

In contrast with Figure 4, Figure 5 shows the optical microscope images for the heat-treated 50 µm and 100 µm layer components. In Figure 5a, representing the heat-treated 50 µm layer product, these microstructures consist of fully recrystallized and irregular (nonequiaxial) grains having dimensions as small as 2 µm and as large as ~1 mm. There is some slight tendency for grains to orient in the build direction, but certainly, there is no preferred orientation. Essentially, all of the grains contain annealing twins of varying sizes; coincident with fcc {111} planes. It is universally recognized that the straight boundary traces for annealing twins in fcc alloys are coincident with {111} planes [20,21,22,23,24,25,26,27,28,29]. It is also notable that the grain boundaries contain continuous carbide segregation while the {111} twin boundaries (the straight boundary traces) do not. On the other hand, close examination reveals that the noncoherent steps and the ends of the twins contain carbides. It might also be noted that the as-built, columnar, <001> textured grains (Figure 4), also do not exhibit annealing twin formation. These phenomena result from interfacial free energy considerations, especially involving low-stacking fault-free energy [20] and the net effect on internal (local) energy; although the formation of annealing twins in fcc metals and alloys remains a controversial issue after more than a half-century [20,21,22,23,24]. Figure 6a shows an enlarged view of these interrelated phenomena in Figure 5a. Carbides are also observed within the grains in all heat-treated cases.

It should be noted that while Figure 5b shows a recrystallized grain structure for the heat-treated, 100 µm layer-built Inconel 625 product, it also shows a duplex grain structure composed of islands of small, equiaxed grains, having an average grain size of ~35 µm, with no annealing twins and continuous carbides in the grain boundaries, which etch poorly. These islands, roughly 550 µm in diameter, are dispersed in very large grains (1–1.5 mm) containing grain boundary carbides and annealing twins shown enlarged in Figure 6b. The issue of grain sizes and area fraction for duplex grain structures is treated in ASTM E1181-02 standard. Studies have shown that small grains do not form twins, and there appears to be a critical size below which annealing twins do not form [24,25]. Horiuchi and Satoh [26] have noted that duplex structures as shown in Figure 6b have been investigated for over a half-century [27]. In the case of nickel-based superalloys such as Inconel 690, M_23_C_6_ carbides in the grain boundaries act as grain-growth inhibitors to promote the bimodal grain structure formation [26]. In these structures, the small grain islands contribute to high strength while the large grain matrix contributes to an unusually large elongation. Arora et al. [28] have also recently observed these features for dual-phase bimodal steel through friction-stir processing. The Vickers microindentation hardness (HV) measurements corresponding to the heat-treated 50 µm and 100 µm layer components as shown in Figure 5 and Figure 6 tend to confirm the novel features of the duplex grain structure. A value of HV 185 with a standard deviation of ±4 was measured for the 50 µm heat-treated layer products (Figure 5a and Figure 6a), while a value of HV 282 and a standard deviation of ±82 was measured for the corresponding 100 µm layer products (Figure 5b and Figure 6b). The very large standard deviation reflects the contributions from the small-grain islands and the large-grain matrix. The tensile data, to be discussed below, confirms this novel feature.

The carbide precipitation on grain boundaries and the precipitation selectivity (continuous carbides segregated to the grain boundaries and noncoherent twin boundaries, but not on the {111} coherent twin boundaries) has been well documented for a number of low-stacking fault-free energy fcc alloys, including 304 stainless steel [20,29] and Inconel 690 [30,31]. In the case of 304 stainless steel, the grain boundary free energy, noncoherent twin boundary free energy, and the coherent {111} twin boundary free energy at 1060 °C have been measured to be 835, 209, and 19 mJ/m^2^, respectively [20]. Consequently, the requisite interfacial free energy for carbide nucleation lies between 209 and 19 mJ/m^2^ at this temperature and indicating that precipitation will not form on low-energy grain boundaries. While these energies are not known for Inconel 625, the stacking fault-free energies for 304 stainless steel and Inconel 600 have been given as 21 and 28 mJ/m^2^ at 25 °C [20], suggesting that Inconel 625 would have interfacial free energy profiles similar to 304 stainless steel. While the presence of carbides on grain boundaries is not likely to degrade the mechanical properties, their formation by the depletion of Cr to form Cr_23_C_6_ carbides to grain boundaries promotes sensitization and susceptibility to corrosion and stress-corrosion cracking [20,29,30,31]. However, as noted originally by Watanabe [32], this feature can be reduced or avoided by systematically reducing the grain boundary area while increasing the {111} coherent twin boundary area, a phenomenon described as grain boundary engineering, applied in the development of many low-stacking fault-free energy fcc alloy systems [30,31,33], including additive manufactured systems [31,33,34]. Of course, a reduction in carbon in the component manufacturing process will also reduce the propensity for carbide formation.

### 3.1. Mechanical Property and Microstructure Comparison and Discussion

Table 3 summarizes and compares the measured mechanical (tensile) properties for both the as-built and heat-treated 50 µm and 100 µm layer processed Inconel 625 components fabricated by electron beam powder bed fusion. The previously discussed Vickers microindentation hardness (HV) values and standard deviations are also listed along with density measurements. It should be noted again that the horizontal tensile reference (as shown in Figure 2) represents the tensile axis perpendicular to the build direction (B in Figure 5 and Figure 6), while the vertical tensile reference (Figure 2) represents the tensile axis in the build direction (parallel to the build direction, B). Normally, the vertical reference tensile orientation is used for the mechanical (tensile) properties of AM products, especially laser and electron beam powder bed fusion processing. In addition, tensile properties almost universally indicate anisotropy as evident in the tensile values shown in Table 3 because of the columnar microstructures, although the 100 µm layer as-built components are somewhat anomalous since the vertical reference yield strength and UTS are greater than the horizontal (359 MPA, 664 MPa versus 330 MPa, 643 MPa, respectively). It is particularly notable that in recent laser beam powder bed fusion processing of Inconel 625, as-built component tensile yield strength and elongations were 633 MPa, 12% in the horizontal tensile direction, and 444 MPa, 21% in the vertical direction [34]. This is in contrast with the heat-treated 100 µm layer component yield and elongations of 581 MPa, 65% in the horizontal reference direction, and 489 MPa, 53% in the vertical reference direction (Table 3). Previous electron beam powder bed fusion of Inconel 625 showed values of yield stress and elongation for as-built and HIPed components in the vertical reference direction of 330 MPa and 69% [4].

It is also notable in Table 3 that the large variance for the microindentation hardness observed for the 100 µm layer heat-treated components is also a characteristic of the corresponding tensile properties, where for the horizontal reference, the yield strength and UTS exhibit a standard deviation of ± 31 and 40, respectively, in contrast with ±3 for the 50 µm layer heat-treated components. This feature is even greater for the vertical reference direction where in Table 3 the standard deviations are ± 176 and 57, respectively, for the yield stress and UTS. As noted previously, this feature results from the bimodal grain structure.

The high strength (581 MPa) and ductility (~65% elongation) along with the microindentation hardness of HV 282 observed for the heat-treatment-induced duplex grain structure in the 100 µm layer products (Figure 5 and Figure 6) is in contrast with the as-built 100 µm product (Figure 4b), where the yield stress was 330 MPa and the ductility (elongation) was 28%; with a corresponding microindentation hardness (HV) of HV 220. This is a unique characteristic of duplex grain structures as previously noted [26,27,28].

### 3.2. Fracture Surface Observations and Discussion

**Figure 7:**
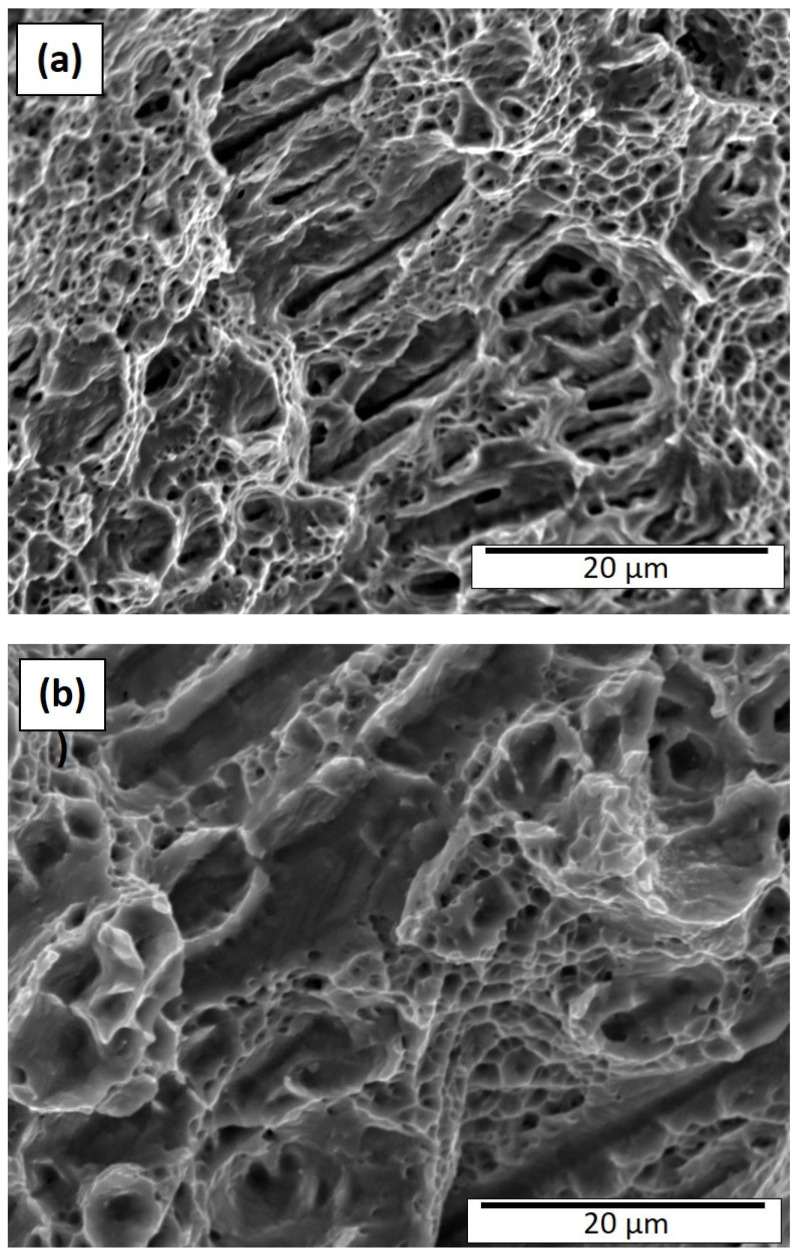
As-built fracture surface (SEM). (**a**) 50 µm layer thickness; (**b**) 100 µm layer thickness.

It is worth noting that the fracture surface features for the as-built and heat-treated Inconel 625 components were fully compatible with the corresponding microstructures and grain structures shown in Figure 4, Figure 5 and Figure 6, as well as the associated mechanical properties for the horizontal tensile geometries summarized in Table 3. Figure 7a,b show SEM fractographs for the 50 µm and 100 µm layer as-built components. The ductile dimple features are very inhomogeneous and include dimple sizes ranging from ~0.4 to >8 µm, with channel-like dimple features having linear cracks. These features, ~25 µm in length, ~2–3 µm wide, and parallel to the build direction, presumably occur at the dense precipitate arrays as shown in Figure 4a and Figure 7b. On the other hand, these features show more diffuse but inhomogeneous dimple features which reflect the lower density of precipitate columns shown in Figure 4b. These features are not characteristic of the fracture surface for the more standard vertical tensile geometries where the dimple structure is homogeneous [4]. However, Anam [35] and Gonzales et al. [36] have also recently illustrated inhomogeneous and linear dimple structures in the build direction for Inconel 625 tensile samples fractured in the horizontal direction (perpendicular to the build direction) as in Figure 7. It is also interesting that the overall smaller dimple features in Figure 7a in contrast with Figure 7b are generally consistent with the corresponding yield stress values shown in Table 3: 387 MPa versus 330 MPa, respectively. The elongations are similarly correlated: 42% versus 28%, respectively.

**Figure 8:**
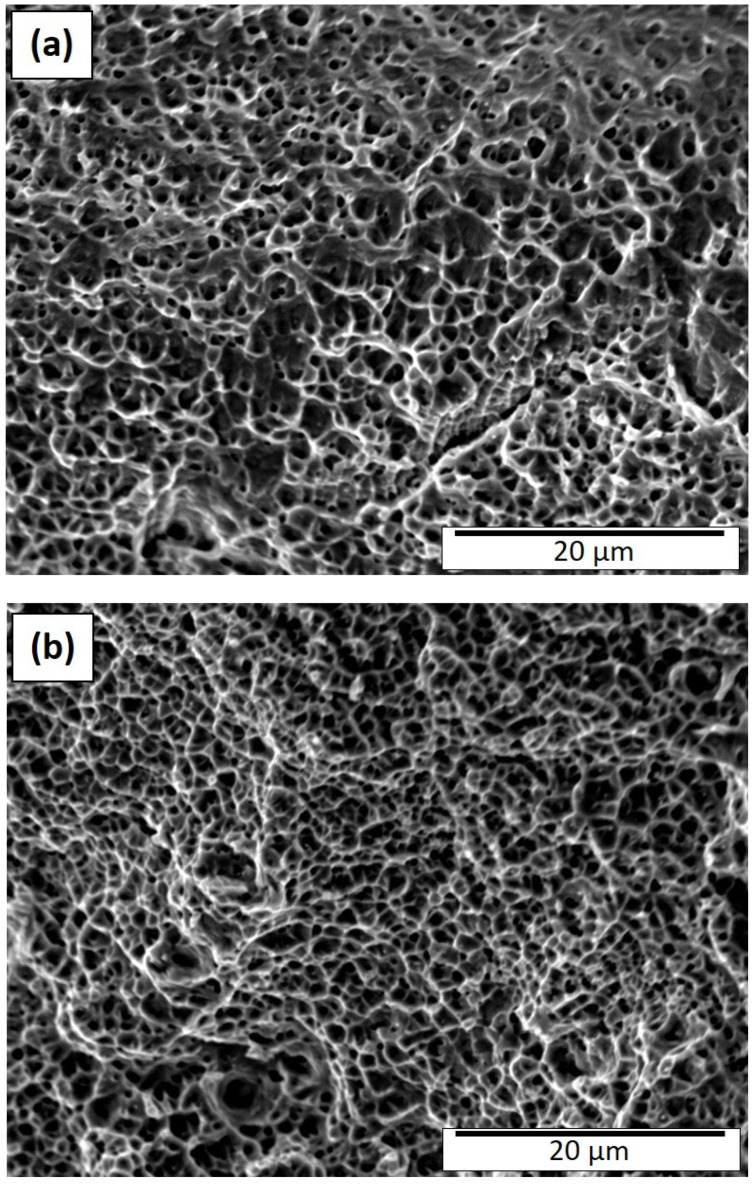
Heat-treated fracture surface (SEM). (**a**) 50 µm layer thickness; (**b**) 100 µm layer thickness.

The ductile fracture features shown in Figure 8a,b for the heat-treated 50 µm and 100 µm layer built components are more homogeneous and characteristic of regular grain structures. Dimple sizes in Figure 8a range from ~0.6 to 3 µm while those in Figure 8b range from ~0.4 to 4 µm, although the average dimple sizes were ~2 µm and 1.5 µm, respectively; the latter including the duplex grain structure which tends to average over the fracture surface. Simply looking closely at the images in Figure 8 can allow a correlation between the corresponding yield stress values of 340 MPa and 581 MPa, respectively, as shown in Table 3. The very similar elongations characteristic of Figure 8a,b of 64% and 65%, respectively, also attest to the dimple similarities. Aside from the somewhat anomalous behavior for the duplex grain structure represented in Figure 5b and Figure 8b, the fracture features generally follow those for a range of alloys where overall dimple size increases when the yield stress decreases [37,38,39].

## 4. Summary and Conclusions

This study compared the microstructures and corresponding hardness (HV) and tensile properties for as-built and post-heat-treated (>1100 °C) 50 µm and 100 µm layer thickness Inconel 625 alloy components fabricated by electron beam powder bed fusion additive manufacturing. The as-built components exhibited lower microindentation hardness and decreased yield stress for the 100 µm layer thickness samples in contrast with the 50 µm layer thickness samples. Both layer thickness components were characterized by columnar grains containing discontinuous columns of precipitates (Ni_3_Nb) extended in the build direction. In contrast, the heat-treated 50 µm layer thickness components exhibited a recrystallized, non-equiaxed grain structure containing fcc {111} coherent annealing twins, with continuous carbide (Cr_23_C_6_) segregation to the grain boundaries and within the grains but not the coherent {111} twin boundaries. The 100 µm layer thickness components exhibited a duplex/bimodal grain structure composed of islands of small equiaxed grains dispersed in a matrix of large grains containing annealing twins. Carbides were continuously segregated to the grain boundaries and within the grains. This grain structure produced a high yield stress which increased by 76% from the as-built 100 µm layer thickness components; with an accompanying high ductility, characterized by an elongation increase of 132% from the as-built 100 µm layer thickness components. These findings led to the following specific conclusions:

- Thicker layer builds (100 µm layer thickness) for electron beam powder bed fusion fabrication of Inconel 625 alloy produce reduced yield stress and elongation in contrast with the 50 µm layer built components.
- High-temperature heat treatment of Inconel 625 alloy components built using 50 µm and 100 µm layer thicknesses produced a recrystallized, non-equiaxed grain structure containing {111} annealing twins, with continuous carbide segregation to the grain boundaries but not the coherent {111} twin boundaries. In contrast, the 100 µm layer thickness heat-treated components produced a duplex grain structure consisting of islands of small equiaxed grains dispersed in a matrix of large grains containing {111} annealing twins, with continuous carbide segregation to the grain boundaries.
- The most significant observation in this study was that the tensile yield strength for the 100 µm layer thickness as-built components increased by 76% following heat treatment, along with an increase of 132% for the corresponding elongation. This unusual development in the residual mechanical properties results from the duplex grain structure where the small grain islands control the yield strength while the elongation (ductility) resides in the large grain matrix.
- High-temperature heat treatment of electron beam powder bed fusion fabricated Inconel 625 alloy can have rather dramatic effects on the residual mechanical properties, including hardness, especially the prospects for producing high strength with accompanying high ductility.
- Therefore, thicker layer fabrication of Inconel 625 alloy by electron beam powder bed fusion provides unusual mechanical property advantages along with additive manufacturing layer building efficiency.

## Figures and Tables

**Table 1:** Chemical composition of Inconel 625 from Praxair.

Element	Composition
Aluminum	0.40 Max
Boron	0.006 Max
Carbon	0.05 Max
Cobalt	1.0 Max
Chromium	20.0–23.0
Copper	0.05 Max
Iron	5.0 Max
Manganese	0.10 Max
Molybdenum	8.0–10.0
Nitrogen	0.025 Max
Nb (Cb)	3.15–4.15
Nb (Cb) + Ta	3.15–4.15
Nickel	Balance
Oxygen	0.03 Max
Phosphorous	0.015 Max
Sulfur	0.015 Max
Selenium	0.005 Max
Silicon	0.50 Max
Tin	0.01 Max
Tantalum	0.20 Max
Titanium	0.40 Max

**Table 2:** EBM printing parameters.

EBM Printing Parameters	
Preheat	Focus Offset = 150 mA
Preheating 1	Max. Current = 30 mA
Preheating 2	Repetitions = 8
	Average Current = 13 mA
Melt-Contours	Num. of Contours = 2
Contours-Outer	Spots = 50
	Spot Time = 1 ms
Beam	Focus Offset = 25 mA
	Speed Function = 40
	Manual Current = 14 mA
Hatch	Line Offset = 0.2 mm

**Table 3:** Average mechanical properties of as-built parts and their corresponding (±) standard deviation; hardness (HV) and density measurements.

		Yield Strength at 0.2% Offset (MPa)	Ultimate Tensile Strength (UTS) (MPa)	Elongation at Fracture (%)
As built (50 μm) ^a^	Horizontal ^b^	387 ± 4	767 ± 18	42 ± 8
	Vertical	365 ± 5	710 ± 10	53 ± 10
As built (100 μm)	Horizontal	330 ± 10	643 ± 111	28 ± 14
	Vertical	359 ± 9	664 ± 7	33 ± 11
Heat Treated (50 μm)	Horizontal	340 ± 3	799 ± 3	64 ± 1
	Vertical	321 ± 2	731 ± 4	56 ± 13
Heat Treated (100 μm)	Horizontal	581 ± 31	711 ± 40	64.8 ± 7
	Vertical	489 ± 176	636 ± 57	53 ± 7
**Hardness and Density Measurements**				
	**Hardness (HV)**	**Density (g/cm^3^)**	**Density** **(%)**	
As Built (50 μm) ^a^	227 ± 12	8.42	99.78	
As Built (100 μm)	220 ± 9	8.39	99.40	
Heat Treated (50 μm)	185 ± 4	8.40	99.54	
Heat Treated (100 μm)	282 ± 82	8.39	99.40	

^a^ Layer building thickness (t). ^b^ Tensile testing direction: horizontal, perpendicular to the build direction (Figure 2); vertical, parallel to the build direction (Figure 2).

## Data Availability

Not applicable.
